# Surgical stoma recurrence after total laringectomy

**DOI:** 10.1016/S1808-8694(15)31127-7

**Published:** 2015-10-20

**Authors:** Andre Luis Sartini, Antonio Sérgio Fava, Pablo Henrique de Faria

**Affiliations:** 1 M.S Student. Assistant physician – Otorhinolaryngology/Head and Neck Surgery Department of the Hospital do Servidor Público Estadual de São Paulo; 2 PhD in Surgery – School of Medicine - USP, FMUSP, In charge of the Head and Neck Surgery Department at the ENT-HNS - Hospital do Servidor Público Estadual de São Paulo; 3 Former Resident Physician at the Otorhinolaryngology/Head and Neck Surgery Department of the Hospital do Servidor Público Estadual de São Paulo. MD. Otorhinolaryngologist

**Keywords:** stoma recurrence

## Abstract

**Summary:**

Stoma recurrence after total laryngectomy is one of the most severe developments of squamous cell carcinoma of the larynx. Risk factors most strongly implicated in stoma recurrence have been subglottic invasion by the laryngeal tumor and tracheotomy prior to laryngectomy.

**Aim:**

Study the clinical findings of patients who underwent total laryngectomy and evaluate the probable risk factors to the development of stoma recurrence.

**Study design:**

Descriptive and retrospective study

**Materials and Methods:**

We studied data from 47 patients who underwent total laryngectomy for the treatment of laryngeal cancer between 1995 and 2004 and evaluated recurrences and risk factors.

**Results:**

Stoma recurrence developed in 10.6 per cent of them(5 cases). There was no significant correlation between stoma recurrence and subglottic invasion or prior tracheotomy.

**Conclusion:**

Stoma recurrence still is one of the most lethal developments associated to laryngeal cancer. In the present study it was not possible to identify factors related to this recurrence. Further studies with a larger sample and a longer follow-up period are necessary to better understand this condition.

## INTRODUCTION

Tumor recurrence in the ultimate stomal area after total laryngectomy is one of the most severe developments of the larynx epidermoid carcinoma treated surgically.

Recurrences in the tracheostoma area started to be better studied after the study by Keim et al.^1^ in 1965. These authors defined it as diffuse neoplastic infiltrations in the area where the trachea stump joins the skin.

According to numerous studies, recurrence incidence in the tracheostoma area after total laryngectomy varies from 1.7% to 25% of laryngectomized patients. Nonetheless, there is great difficulty in knowing the real value of such recurrence rates, since many papers include them in the local recurrence group^2^,^3^.

Another significant factor that may cause difficulties in analyzing recurrence incidence in the tracheostoma area is the number of synonyms used to describe such pathology, such as: tracheal metastasis, peritracheal recurrence, tracheal recurrence, paratracheostomal tumor, tracheostomal metastasis, second primary tumor in the trachea^3^.

Most relapses in the tracheostoma area are diagnosed in the first year after total laryngectomy and its pathogeny is still not clearly defined^1^,^4^, ^5^, ^6^, ^7^.

Tumor site and disease staging, infraglottic invasion, previous tracheostomy, insufficient surgical margins after total laryngectomy, thyroid invasion by the laryngeal tumor, tumoral implants during surgery and paratracheal lymphnode metastasis are the most mentioned correlated factors in the literature^1^, ^2^, ^3^, ^4^, ^5^, ^6^, ^7^, ^8^, ^9^, ^10^, ^11^, ^12^, ^13^, ^14^, ^15^, ^16^, ^17^, ^18^, ^19^, ^20^, ^21^, ^22^, ^23^, ^24^, ^25^, ^26^. There is no unanimous opinion as to the importance of these factors in tumor recurrence in the tracheostoma area.

The different treatment modalities for these recurrences, that include surgery, radio and chemotherapy, have not been satisfactory to control the disease, and therefore, a special attention has been given to the prevention of such pathology^2^,^8^, ^9^, ^10^. Many methods have been proposed to prevent recurrence in the tracheostoma area, such as: lower trachea section allowing for a broader surgical margin, dissection of lymphnodes located in the paratracheal chains, emergency laryngectomies in patients that required tracheostomy prior to their laryngectomy, postoperative radiotherapy including the tracheostoma and the upper mediastinum^1^,^3^,^7^,^11^.

Historically, recurrences in the tracheostoma area are considered very difficult to treat and are associated to little likelihood of survival^10^,^12^, ^13^, ^14^, ^15^.

The goal of the present study is to investigate the cases of patients in our institution who suffered laryngectomy between the years of 1995 and 2004, and analyze the cases in which there was tumor recurrence in the tracheostoma area by means of the clinical findings in these patients, trying to identify some risk factors in the causation of such pathology and what could prevent it.

## MATERIALS AND METHODS

This study is based on 47 patients diagnosed with larynx epidermoid carcinomas, who underwent total laryngectomy, from February of 1995 to January of 2004. All the patients included in this study did not undergo radio or chemotherapy prior to surgery.

As far as gender is concerned, males predominated (44 cases), when compared to females (3 cases), bearing a 14,6:1 ratio.

Minimum age was of 42 years and maximum age was 86 years. Average age was 63.3 years and the median was 61 years.

The surgical approach was total laryngectomy, including the resection of the tracheostomy area in those patients who had been tracheostomized prior to surgery, either with or without radical or selective uni or bilateral neck clearance. Indication for postoperative radiotherapy was determined for each patient after the results from the pathology study, and it was indicated in the cases of compromised surgical margins or the presence of histologically metastatic neck lymphnodes.

The lesions were classified according to the TNM-UICC classification method. In order to frame the cases in specific groups, we considered the whole set of data from the nasofibroscopy exam, direct and indirect laryngoscopy, neck palpation, neck CT scan and chest X-rays.

Histopathologic diagnosis was carried out by means of biopsies that were, most of them, done under direct laryngoscopy and general anesthesia. In cases of dyspneic patients at the time of clinical diagnosis, they were submitted to emergency tracheostomy under sedation and local anesthesia in the operating theater and, later on, direct laryngoscopy under general anesthesia.

In order to carry out the statistical analysis among the different qualitative variables from the 47 patients who underwent total laryngectomy we used the Chi-Squared test or the Fisher Exact test, when necessary, aiming at identifying possible relations among variable classes and the groups of patients that either presented or did not present lapses in the area of the tracheostoma. We used the Mann-Whitney non-parametric test in order to compare ages among the groups of patients with and without recurrence in the tracheostoma area.

The tests were carried out considering a 5% significance level.

The variables analyzed were the following: age, gender, clinical staging (TNM), tumor location, previous/preoperative tracheostomy, postoperative radiotherapy.

## RESULTS

Recurrences in the tracheostoma area occurred in 5 cases (10. 6%) of 47 patients who underwent total laryngectomy.

There was no statistically significant difference among ages (p=0.449) and gender (p<0.999) of the patients who had recurrences in the tracheostoma area when compared to those who did not have such recurrence.

Among the 47 laryngectomized patients, according to the TNM-UICC classification, 14 (29.78%) patients were staged as T2; 23 patients (48.9%) as T3 and 10 (21.27%) patients were T4 (Table 1).

**Table 1:** Recurrence in the tracheostoma area according to clinical characteristics.

Characteristics	# of patients (%)	# of recurrences in the tracheostoma area	p
Tumor site			0,289
glottis	18 (38,3%)	2	
glottis/infraglottic	4 (8,5%)	2	
supraglottis	3 (6,4%)	0	
supraglottis/glottis	20 (42,6%)	1	
supraglottis/glottis/infraglottic	2 (4,3%)	0	
Previous tracheostomy			0,336
Yes	17 (36,2%)	3	
No	30 (63,8%)	2	
N (clin)			0,240
N0	26 (55,3%)	4	
N1 - N3	21 (44,7%)	1	
T (clin)			0,864
T2	14 (28,8%)	2	
T3	23 (48,9%)	2	
T4	10 (21,3%)	1	
Postoperative radiation			<0,999
Yes	32 (68,1%)	4	
No	15 (31,9%)	1	

* clin – TNM Clinical staging

Of the patients who had recurrences in the tracheostoma area, 2 (40%) had been staged as T2, 2 (20%) patients as T3; and 1 (20%) as T4. There was no statistical significance between tumor size (T) and recurrence in the tracheostoma area (p=0.864) (Table 1).

Analyzing the 47 patients with larynx epidermoid carcinoma who underwent total laryngectomy, 26 (55.31%) did not have palpable neck lymphnode at the time of disease diagnosis, while 21 (44.68%) had clinically palpable lymphnodes and with metastatic characteristic at the time of disease clinical diagnosis (Table 1). There was no statistically significant correlation between neck lymphnode metastasis and tumor recurrence at the tracheostoma area (p=0.240).

The division by compromised structure showed that of the 5 patients with recurrence at the tracheostoma area, 2 (40%) had initial lesion in the glottis only, 2 (40%) had lesion in the glottis/subglottis; and 1 (20%) had lesion in the supraglottis/glottis (Table 2).

**Table 2:** Detailed information on the 5 patients with recurrences in the tracheostoma area

Patient	Age	Primary site	Previous tracheostomy	Postoperative radiation	Staging
1	71	glottis/subglottis	Yes	Yes	T2N0M0
2	58	supraglottis/glottis	Yes	Yes	T2N0M0
3	76	glottis	No	Yes	T3N1M0
4	59	glottis/subglottis	Yes	Yes	T4N0M0
5	70	glottis	No	No	T3N0M0

In our series there was no statistical association between recurrence in the tracheostoma area and the primary tumor affecting the subglottis (p=0.054).

We observed that of the 47 patients who underwent total laryngectomy, 17 (36.17%) had been submitted to tracheostomy prior to the definitive surgery, probably due to dyspnea or tumor size seen at the time of diagnosis. Among the 5 cases of recurrences at the tracheostoma area, 3 (60%) had had previous tracheostomy (Table 2). Even then there was no statistic significance between this variable and recurrences (Table 1).

Postoperative radiotherapy was used in 4 (80%) patients that later on evolved with recurrences at the tracheostoma site. In these patients, radiotherapy was employed because of histologically metastatic lymphnodes in the neck.

We did not observe statistically significant differences when we analyzed the recurrence in the tracheostoma area in relation to the use of postoperative radiotherapy (p<0.999).

## DISCUSSION

Recurrence in the tracheostoma area is considered the most severe and fatal complication of laryngeal cancer. In the many series, the recurrence incidence in the tracheostoma area varies between 1.7% and 25%^2^,^3^, and in our study we report an incidence of 10.6%.

In 1965, Keim et al.^1^ revised 116 cases of patients who underwent total laryngectomy because of larynx epidermoid cancer. Recurrences in the tracheostoma area happened to 70 patients, all of them died. It was then defined that the concept of recurrence in the tracheostoma area would be a diffuse neoplastic infiltration, at the junction between the trachea stump and the skin. Based on such criteria, the lesion could compromise the stomal epithelium, adjacent tissue or both.

The true rates are unknown because many series reported in the literature classify the recurrence in the tracheostoma area as part of local recurrences^3^.

Another problem found is the large number of synonyms used to define this type of pathology, such as: tracheal metastasis, peritracheal recurrence, tracheal recurrence, paratracheostomal tumor persistence, tracheostomal metastasis, and second primary tumor in the trachea^3^.

Most of these recurrences are diagnosed in the first year after total laryngectomy12. Kowalski16 reported an average of 5.5 months for recurrence to appear. In his study, 19 of 24 patients (79.2%) had recurrence after 1 year of follow up. Contrasting that, Modlin and Ogura^11^ reported a longer period for the recurrence diagnosis, varying from one to two years after laryngectomy.

The tumor primary site is a significant risk factor for recurrence in the tracheostoma area. In larynx cancer, infraglottic involvement remains as the major risk factor for recurrences in the tracheostoma area^1^,^6^,^14^,^18^. Many authors believe that these tumors have a tendency towards fast paratracheal tissue involvement, because of its submucosal expansion. There also is a natural trend for infraglottic tumors to infiltrate the larynx framework and consequently progress to invade the thyroid and perilaryngeal soft tissue^19^, ^20^, ^21^, ^22^. In patients whom the infraglottic region was involved, the recurrence rate in the tracheostoma region may reach 38%6,15 and 44%7. In our study, the Infraglottic region involvement presented a statistical significant trend, and a p descriptive value of 0.054.

As far as the neck is concerned, although we have not seen statistically significant difference among patients clinically NO and N1-3 in the genesis of recurrence in the tracheostoma area in our study (p=0.240), we have to stress the importance of paratracheal lymphnodes in larynx cancer reported by other authors, especially when the tumor involves the infraglottic region^4^,^6^,^13^,^23^.

Lymphnode metastasis in head and neck tumors is the most important prognostic factor related to patient survival, especially when the primary tumor is located in the larynx, hypopharynx or upper esophagus. Although the lack of prospective studies is responsible for us not having enough information about the ideal surgical approach in the NO neck, elective neck dissection is always advocated when the risk of microscopic lymphnode disease in the neck exceeds 20%^16^.

In larynx tumors, especially those that affect the infraglottic region, the lymphnode group at risk would be the paratracheal and pre-tracheal lymphnode chain, and it is possible if these are not properly investigated and treated, that they would be involved in the recurrence we see in the tracheostoma area^10^,^24^,^25^. These lymphnodes are rarely palpable and are not routinely included in neck dissections. Nonetheless, considering the possibilities of disease dissemination towards this area, it is advisable to carry out neck dissection in these lymphnode chains^16^.

Some authors report a recurrence reduction in the tracheostoma area from 11.5% to 2.7% in laryngectomized patients in whom there was paratracheal and retrosternal dissection together with postoperative radiotherapy involving the upper mediastinum, tracheostoma and paratracheal region.18 Paratracheal neck dissection may show up to 65% of microscopic lymphnode involvement in cases of subglottic involvement.^25^.

Patients with advanced larynx tumors who present dyspnea at the time of diagnosis may require emergency tracheostomy. Many of these tumors that cause upper airway obstruction also extend towards the subglottis. Prior tracheostomy in these patients, necessary to establish a new airway has been identified as an important risk factor for recurrences in the tracheostoma area^1^,^7^,^19^. This is due to the fact that prior tracheostomy may allow the implantation of viable neoplastic cells from tumor shedding that have an intense inflammatory process and repair granulation tissue - the bed may be prone to cell adhesion.2 In the literature such risk is reported as varying between 8 and 41%^1^,^6^.

Many authors also consent that the time span between the tracheostomy and laryngectomy could increase the risk of tumor implantation, thus making the surgical area prone to recurrences.^2^,^5^,^10^.

In an attempt to establish a more adequate prognosis for patients with recurrences in the tracheostoma area, Sisson et al.^12^ described a classification system for recurrences. Type I would be localized recurrence with a small nodule in the upper portion of the tracheostoma, and no esophageal disease. Prognosis is relatively favorable if the recurrence is detected at this stage.

Type II involves the tracheostoma upper half and the esophagus - prognosis is variable, depending on the esophagus region involved. Type III starts in the tracheostoma lower half, and would usually have a direct invasion towards the mediastinum. Type IV indicates that the disease extends laterally, involving the region below the clavicles.

Another risk factor proposed for recurrences in the tracheostoma area would be endotracheal intubation as a source of transport and implantation of tumor cells^20^.

Other authors also describe recurrences in the tracheostoma area in patients subjected to total laryngectomy that had previously undergone partial laryngectomy^5^,^10^.

It has been suggested that possible tumor cell implants could occur in the tracheostoma area through the very contact of this area with the surgeon's hand, that very likely moments before were manipulating the primary tumor area. We believe and agree with these authors that tumor implantation must not be totally disregarded as a possible mechanism of remote tumor spread.

Reddy et al.^21^ investigated the relationship of p53 elevation and recurrences in the tracheostoma area, when they studied a group of patients classified as T1 glottic. These patients were initially treated by radiotherapy alone and subsequently submitted to rescue laryngectomy because of local tumor recurrence. Recurrence in the tracheostoma area happened in 25% of the patients who underwent rescue laryngectomy after the first recurrence. There was no correlation between p53 elevation, obtained by means of an immunohistochemistry analysis of a larynx biopsy before radiotherapy and recurrence in the tracheostoma area.

Identifying tumor markers related to the risk of distant spread, as well as chemo or radiosensibility would be of great help in planning therapy. Moreover, this could allow us to better understand the disease and then develop new and early methods to detect and treat this disease.

Many prevention methods have been proposed in an attempt to preclude recurrences in the tracheostoma area, such as lower trachea resection and paratracheal lymphnode dissection; avoid tracheostomy prior to the definitive surgical procedure; emergency laryngectomy instead of tracheostomy in patients with severe respiratory obstruction; post-operative radiotherapy in the tracheostoma area and upper mediastinum^26^, ^27^, ^28^, ^29^.

As we said before, prior tracheostomy is considered an important risk factor to the development of recurrences in the tracheostoma area. Aiming at avoiding this type of procedure, some authors have proposed emergency laryngectomy in patients with severe respiratory obstruction due to advanced larynx tumors^11^,^24^.

Narula et al.^22^ described the term emergency laryngectomy as being a total laryngectomy carried out within 24 hours of the diagnosis of a malignant larynx neoplasia without previous treatment, because of clinical symptoms of respiratory obstruction presented by the patient with advanced stage larynx cancer. They conclude with this study, in which they compared two random groups (one group submitted to tracheostomy prior to laryngectomy and another group submitted to emergency laryngectomy), that emergency laryngectomy did not offer any advantage for patient survival.

We agree with these authors, because we think the difficulty in performing a proper preoperative evaluation for such a complex procedure and the need to blindly trust the frozen section exam for surgery consideration would be factors that could discourage this type of procedure.

Some papers state that there is a high incidence of thyroid gland involvement in patients with advanced stage larynx epidermoid cancer or with subglottis involvement. Based on these results, the authors advocate partial or total thyroidectomy in these cases^13^,^23^.

Direct thyroid gland invasion may occur in transglottic tumors or supraglottis carcinomas because of tumor invasion through the thyroid cartilage or the cricothyroid membrane^24^,^25^. Indirect or secondary thyroid gland involvement may also occur in subglottis or transglottic tumors because of lymphatic spread to the pre-laryngeal or Delphian node. All these examples mentioned, and if not considered during surgery may, with time, create unexpected tumor recurrence in the tracheostoma area in laryngectomized patients^23^.

Total laryngectomies are usually carried out in a broad field, including pre-laryngeal muscles and the adjacent soft tissue. We deem important the resection of the space between thyroid gland isthmus and the larynx, a place that may be the seat of metastasis (pre-cricothyroid nodes).

We usually do not include the thyroid gland in the surgical specimen. When there is any suspicion of thyroid involvement, we carry out ipsilateral lobectomy on the lesion site or total thyroidectomy.

Better results of tumor recurrence control at the tracheostoma area were obtained through the association of post-operative radiotherapy^27^,^30^, ^31^, ^32^.

We indicate postoperative radiotherapy in cases where the surgical margins were compromised or very thin, and also in the presence of histologically confirmed lymphnode metastasis. In the present study, among the 5 patients who presented recurrences in the tracheostoma area, 4 (80%) had undergone postoperative radiotherapy, including the tracheostomal field. This may cause doubts as to the possible usefulness of postoperative radiotherapy in preventing recurrences in the tracheostoma area.

The treatment of recurrences in the tracheostoma area is not often curative, and palliative treatment results are frequently unsatisfactory^1^,^30^,^33^.

Since the 60's, recurrent tumor and trachea resections associated to upper mediastinum clearance have been the only efficient therapeutic approaches to recurrences in the tracheostoma area. Many authors describe that the best approach for recurrences in the tracheostoma area is surgery^2^,^12^,^28^. Nonetheless, surgery must be customized for each patient, assessing clinical conditions in order to decide for such a complex procedure, and also consider the possible radical resection of this recurrence.

Results from radiotherapy alone are not satisfactory to treat recurrences in the tracheostoma area; nonetheless it is indicated as a palliative treatment in inoperable cases, both due to recurrence unresectability in the tracheostoma area, and unfavorable patient clinical condition^4^,^29^,^32^.

Chemotherapy alone, or associated with radiotherapy do not bring about significant responses, either objective or subjective, and not even enhances patient's life quality for those with extensive and unresectable recurrencies^2^,^16^.

As we see it, surgery is the treatment of choice, depending on recurrence extension and the patient's clinical situation. In our 5 cases, treatment employed was radiotherapy due to recurrence staging and the unfavorable clinical conditions of our patients at the time.

## CONCLUSION

Recurrence in the tracheostoma area after total laryngectomy still is one of the most severe and almost lethal complications of larynx cancer. In the present investigation, we did not identify factors related to the genesis of such recurrence based on the clinical exam of the laryngectomized patients. We need further studies with more cases, longer follow up periods and complementary data from pathology in order to better understand this pathology and identify possible candidates for this type of recurrence and, thus, provide the best treatment possible for them.

## References

[1] Keim WF, Shapiro MJ, Rosin HD (1965). Study of postlaryngectomy stomal recurrence.. Arch Otolaryngol.

[2] León X, Quer M, Burguês J, Abelló P, Vega M, de Andrés L (1996). Prevention of stomal recurrence.. Head and Neck.

[3] Yotakis J, Davris S, Kontozoglou T, Adamopoulos G (1996). Evaluation of risk factors for stomal recurrence after total laryngectomy.. Clin Otolaryngol.

[4] Mantravadi R, Katz AM, Skolnik EM, Becker S, Freehling DJ, Friedman M (1981). Stomal recurrence.. A critical analysis of risk factors. Arch Otolaryngol.

[5] Stell PM, Van den Broek P (1971). Stomal recurrence after laryngectomy: aetiology and management.. J Laryngol Otol.

[6] Bonneau RA, Lehman RH (1975). Stomal recurrence following laryngectomy.. Arch Otolaryngol.

[7] Esteban F, Moreno JA, Delgado-Rodriguez M., Mochon A (1993). Risk factors involved in stomal recurrence following laryngectomy.. J Laryngol Otol.

[8] Imauchi Y, Ito K, Takasago E, Nibu K-I, Sugasawa M, Ichimura K (2002). Stomal recurrence after laryngectomy for scamous cell carcinoma of the larynx.. Otolaryngol Head Neck Surg.

[9] Bignardi L, Gavioli C, Staffieri A (1983). Tracheostomal recurrences after laryngectomy.. Arch Otorhinolaryngol.

[10] Rockley TJ, Powell J, Robin PE, Reid AP (1991). Post-laryngectomy stomal recurrence: tumor implantation or paratracheal lymphatic metastasis?. Clin Otolaryngol.

[11] Modlin B, Ogura JH (1969). Post-laryngectomy tracheal stomal recurrences.. Laryngoscope.

[12] Sisson GA, Bytell DE, Edison BD, Yeh S (1975). Transsternal radical neck dissection for control of stomal recurrence: end results.. Laryngoscope.

[13] Biel MA, Maisel RH (1985). Indications for performing hemithyroidectomy for tumors requiring total laryngectomy.. Am J Surg.

[14] Myers EM, Ogura JH (1979). Stomal recurrences: a clinicopathological analysis and protocol for future management.. Laryngoscope.

[15] Weisman RA, Colman M, Ward PA (1979). Stomal recurrence following laryngectomy.. A critical evaluation. Ann Otol.

[16] Kowalski LP (1986). Recidiva de carcinomas de laringe e da parte laríngea da faringe na área do traqueostoma: Análise dos fatores de risco e do tratamento [Tese de Mestrado].

[17] Halfpenny W, McFurk M (2001). Stomal recurrence following temporary tracheostomy.. J Laryngol Otol.

[18] Hosal IN, Onerci M, Turan E (1993). Peristomal recurrence.. Am J Otolaryngol.

[19] Kuehn PG, Tennant R (1971). Surgical treatment of stomal recurrences in cancer of larynx.. Am J Surg.

[20] Ormerod FC, Shaw HJ (1952). An account of morbidity and mortality associated with total laryngectomy.. J Laryngol Otol.

[21] Reddy SP, Marayama A, Melian E, Kathuria S, Leman C, Emami B (2001). Stomal recurrence in patients with T1 glottic cancer after salvage laringectomy for radiotherapy failures:role of p 53 expression and subglottic extension.. Am J Clin Oncol.

[22] Narula AA, Shepard IJ, West K, Bradley PJ (1993). Is emergency laryngectomy a waste of time?. Am J Otolaryngol.

[23] Bahadur S, Iyer S, Kacher SK (1985). The thyroid gland in the management of carcinoma of the larynx and laryngopharynx.. J Laryngol Otol.

[24] Harrison DFN (1971). The pathology and management of subglottic cancer.. Ann Otol.

[25] Harrison DFN (1975). Laryngectomy for subglottic lesions.. Laryngoscope.

[26] Weber RS, Marbel J, Smith P, Hankins P, Goepfert H (1993). Paratracheal lymph node dissection for carcinoma of the larynx, hypofarynx, and cervical esophagus.. Otolaryngol Head Neck Surg.

[27] Tong D, Moss WT, Stevens KR (1977). Elective Irradiation of the lower cervical region in patients at high risk for recurrent cancer at the tracheal stoma.. Radiology.

[28] Sisson GA (1989). 1989 Ogura Memorial Lecture: Mediastinal dissection.. Laryngoscope.

[29] Schneider JJ, Lindberg RD, Jessé RH (1975). Prevention of tracheal stoma recurrences after total laryngectomy by postoperative irradiation.. J Surg Oncol.

[30] Gluckman JL, Hamaker RC, Weissler MC, Schüller DE, Glenwood AC (1987). Surgical salvage for stomal recurrence: A multi-institutional experience.. Laryngoscope.

[31] Barr GD, Robertson AG, Lui KC (1990). Stomal Recurrence: A separate entity?. J Surg Oncol.

[32] Mirimanoff RO, Wang CC, Doppke KP (1985). Combined surgery and postoperative radiation therapy for advanced laryngeal and hypopharyngeal carcinomas.. Int J Radiat Oncology Biol Phys.

[33] Breneman JC, Bradshaw A, Gluckman J, Aron BS (1988). Prevention of stomal recurrence in patients requiring emergency tracheostomy for advanced laryngeal and pharyngeal tumors.. Cancer.

